# Social Media for Marketing Surgical Practice: Instagram Use by German Plastic Surgeons

**DOI:** 10.1097/GOX.0000000000007645

**Published:** 2026-04-23

**Authors:** Johannes C. Heinzel, Derya Durak, Alexander Hönning, Wiebke Käckenmester, Martin Aman, Adrien Daigeler, Jonas Kolbenschlag, Cosima Prahm

**Affiliations:** From the * Department of Hand-, Plastic, Reconstructive and Burn Surgery, BG Unfallklinik Tübingen, Eberhard Karls University, Tübingen, Germany; † Department of Dermatology, University Hospital Tübingen, Eberhard Karls University, Tübingen, Germany; ‡ Center for Clinical Research, BG Klinikum Unfallkrankenhaus, Berlin, Germany; § Department of Hand-, Replantation, and Microsurgery, Charité Universitätsmedizin Berlin, BG Klinikum Unfallkrankenhaus, Berlin, Germany.

## Abstract

**Background:**

Instagram has emerged as a prominent platform for professional engagement in plastic surgery, enabling surgeons to educate, network, and promote their services. The aim of this study is to determine how widespread the professional use of Instagram is among German plastic surgeons and to describe the level of engagement on this platform.

**Methods:**

The professional Instagram use of all 1281 members of the German Society of Plastic, Reconstructive and Aesthetic Surgery (DGPRÄC) was analyzed in March 2022. Publicly identifiable Instagram accounts were assessed for activity metrics, such as the number of posts, followers, accounts followed, and types of content. Instagram use was analyzed in relation to clinical position, work setting, sex, and geographic location using descriptive statistics and χ^2^ tests.

**Results:**

A total of 407 (31.8%) DGPRÄC members used Instagram to post professional content. Instagram use was significantly higher among practicing physicians (47.7%) compared with hospital-based surgeons (8.6%–20.8%, *P* < 0.001). The median number of posts was 65, with a median posting frequency of 1.8 posts per month. The median follower count was 653. Surgeons in city-states and those in private practice were significantly more active on Instagram. Nearly half of all accounts contained both professional and private content.

**Conclusions:**

Instagram is a common professional tool for plastic surgeons, particularly those in private practices and urban areas. Although professional engagement was evident, overall activity remained moderate in terms of post frequency and audience reach.

**Takeaways**

**Question:** How widespread is the professional use of Instagram among German plastic surgeons, and what factors influence engagement?

**Findings:** Among 1281 members of the German Society of Plastic, Reconstructive and Aesthetic Surgery (DGPRÄC), 31.8% used Instagram professionally. Instagram use was significantly more common among practicing physicians (47.7%) than hospital-based surgeons (8.6%–20.8%). Surgeons in private practice and urban regions were more active. Median posting frequency was 1.8 posts per month, with a median of 653 followers.

**Meaning:** Professional use of Instagram among German plastic surgeons is moderate and primarily concentrated in private practice; broader engagement could enhance professional visibility and help address misinformation.

## INTRODUCTION

Instagram is an online social media platform that allows its users to share photographs and videos, interact with other users, and view content from people or organizations. Unlike text-focused platforms such as X (formerly Twitter), Instagram has a strong visual focus. Originally developed to share private photographs, Instagram became an important tool for self-promotion and marketing. The online service has developed into a significant advertising platform that is increasingly used in the medical field.^1^ Due to its strong visual focus, it has been hypothesized that Instagram could play an important role in plastic surgery.^2^ Indeed, the use of Instagram as a communication platform for plastic surgeons has increased significantly in recent years.^3,4^ Among European plastic surgeons, Instagram is one of the most frequently used social media platforms.^4^ The use of Instagram has also increased significantly among patients. For patients considering breast augmentation, Instagram has been the most frequented social media platform to gather information before consulting a surgeon.^5^

For plastic surgeons, the frequent use of social media opens the opportunity to reach a large number of potential patients at a comparatively low cost.^6^ Having a presence on Instagram allows them to share their expertise, reach potential patients, and raise awareness of their services. However, the platform poses risks in terms of the quality of medical information and the ease of spreading misinformation.^7^ For instance, a significant proportion of nonplastic surgeons share content on aesthetic procedures, which poses potential risks to patient safety and the quality of treatment outcomes.^8^ The presence of plastic surgeons on Instagram might therefore be crucial in counteracting misinformation. By increasing their presence on Instagram, plastic surgeons can help to increase the professional representation of their specialty and correct misconceptions.

This study examines the use of Instagram by plastic surgeons in Germany. The aim is to determine how widespread the professional use of Instagram is among plastic surgeons and to describe the level of engagement on this platform.

## METHODS

### Study Design and Sample

A publicly accessible list of the members of the German Society of Plastic, Reconstructive and Aesthetic Surgery (DGPRÄC) was used to identify plastic surgeons who actively practice in Germany. After identifying all active full members of the DGPRÄC, their Instagram accounts were searched on Google using the terms “Instagram” and the respective surname OR first name and surname OR surname and city OR plastic surgery and surname. The search by first name, surname, city, and specialty was then carried out on Instagram. If an account that came up in the search could not be matched, the username of the identified profile was compared with the followers of the DGPRÄC’s official Instagram account. As members with a larger reach on Instagram are easier to find, these followers and subscribed accounts were also checked for other members. Profiles of members who could not be clearly assigned were registered as not using Instagram due to identification difficulties. In March 2022, the identified accounts were examined for their number of posts, followers, and followed accounts.

### Ethics

The study complied with ethical guidelines, using only publicly available information without sensitive personal data. According to the Ethics Committee in Tübingen and the Leibniz Institute for Knowledge Media (confirmation from Prof. Dr. Dr. S. Utz, February 15, 2022), no ethical approval was required. All data were summarized, anonymized, and used solely for statistical analysis, ensuring both data protection and scientific integrity.

### Terminology and Characteristics Examined

#### Followers

People who have subscribed to the account of a DGPRÄC member regularly receive their posts and content. By subscribing to an account, a follower shows a long-term interest in its content and thus receives continuous updates and notifications. The number of followers of an account is often seen as the most important measure of its reach, influence, and popularity. A high follower count can indicate that the account offers interesting and relevant content that appeals to many people.

#### Followed Accounts

Followed accounts are those that the DGPRÄC member follows to see photographs or videos in the Instagram feed. Following other accounts is the main way of connecting with people on Instagram.

#### Clinical Position

A *chief* exercises a managerial function in the hospital department. They are responsible for the professional guidance of the physicians in the department and bear disciplinary responsibility. *Attendings* hold a leadership position in the department. They supervise residents, handle complex treatments or operations, and are responsible for the medical aspects of their department. *Residents* are in the process of completing training within their chosen specialty. *Practicing physicians* offer medical services in their own practice. They are independent and do not work in hospitals or medical facilities.

#### Working Place

*Teaching hospitals* partner with medical and nursing schools, education programs, and research centers to improve health care through learning and research. *Nonteaching hospitals* do not offer educational opportunities for students, nurses, or other medical professionals, but provide essential medical services to the communities they serve. *BG hospitals (Berufsgenossenschaftliche Kliniken*) are the healthcare providers of the statutory accident insurance in Germany and specialize in the treatment and rehabilitation of patients with severe trauma and occupational diseases. In a *medical practice*, 1 or more physicians work, and the range of services is based on the physicians practicing there.

### Statistical Analysis

The statistical analysis was carried out descriptively in an exploratory manner. The sample size (N = 1281) was deemed sufficiently large to gain reliable and meaningful results. Characteristics of DGPRÄC members were summarized with absolute (n) and relative (%) frequencies. Depending on the scale level, the Instagram activity was reported using median values, interquartile ranges (IQRs), minimum and maximum values, and absolute and relative frequencies. Variables of categorical scale level (ie, clinical position, working place, sex, type of federal state) were tested for significant differences with the Pearson χ^2^ test of independence. *P* values less than 0.05 were considered statistically significant. Line charts, bar plots, and box plots were used to visualize Instagram activity and associations between Instagram use and relevant parameters. SPSS version 27.0 (IBM Deutschland GmbH, Ehningen, Germany) was used for data entry; R version 4.1.2 (The R Foundation for Statistical Computing, Vienna, Austria) was used for all statistical analyses. Reporting is carried out in accordance with STROBE principles.

## RESULTS

### Characteristics of the Study Population

As of March 2022, the DGPRÄC had 1281 members, of whom 582 could be identified as having Instagram accounts. Of these, 74 (5.8%) did not post at all, 49 (3.8%) posted only nonprofessional content, 52 (4.1%) had private accounts, and 407 (31.8%) posted professional content on Instagram. The majority of Instagram users posting professional content were men (70.3%) and used it in 1 of the 13 German territorial states (85.8%). Twelve percent headed a hospital department as chiefs, 26% were attending, and 9% were residents. Although approximately half of the plastic surgeons worked as physicians in a medical practice (52.8%), slightly more than one-third worked at a hospital that provides medical education (36.8%), 10.4% worked at a nonteaching hospital, and 4.2% were employed at BG hospitals (Table 1).

**Table 1.** Characteristics of Study Population

Variable	Instagram Use (N = 407)	No Instagram Use (N = 874)	Total (N = 1281)
Sex, n (%)			
Female	115 (28.3)	265 (30.3)	380 (29.7)
Male	292 (71.7)	609 (69.7)	901 (70.3)
Type of federal state, n (%)			
City state	72 (17.7)	110 (12.6)	182 (14.2)
Territorial state	335 (82.3)	764 (87.4)	1099 (85.8)
Clinical position, n (%)			
Chief	32 (7.9)	122 (14.0)	154 (12.0)
Attending	42 (10.3)	291 (33.3)	333 (26.0)
Resident	10 (2.5)	107 (12.2)	117 (9.1)
Practicing physician	323 (79.4)	354 (40.5)	677 (52.8)
Working place, n (%)			
Teaching hospital	62 (15.2)	355 (40.6)	417 (32.5)
Nonteaching hospital	16 (3.9)	117 (13.4)	133 (10.4)
BG hospital	6 (1.5)	48 (5.5)	54 (4.2)
Medical practice	323 (79.4)	354 (40.5)	677 (52.8)

%, proportion of subjects; BG hospital, healthcare provider of the statutory accident insurance.

### Development of Instagram Posts Over Time

**Fig. 1.**
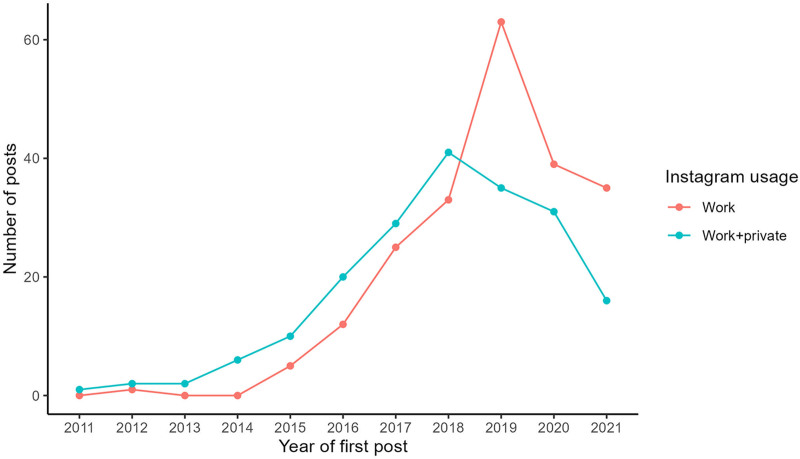
First work-related Instagram posts of plastic surgeons from 2011 to 2021. As only complete years were taken into account, year 2022 is not shown in the graph.

The first work-related Instagram post of a DGPRÄC member was identified in 2011. Although the activity on Instagram remained at a low level until 2014, the number of first posts increased roughly linearly between 2015 and 2018. A notable change occurred in 2019, with initial posts in the group with exclusively work-related content almost doubling (33–63), whereas initial posts in the group with both work-related and private content dropped from 41 to 35. Since 2020, the number of first posts in both groups decreased again, resulting in 35 first posts in the group with exclusively work-related content and 16 first posts in the mixed-content group in 2021. The graph also shows that Instagram accounts were initially used rather for work-related and private content, whereas exclusively work-related content dominated in the later years of the study period (Fig. 1).

### Instagram Activity of DGPRÄC Members

The 407 DGPRÄC members who used Instagram for work-related content posted a total of 54,758 times ranging from 1 to 3657 posts between November 2011 and March 2022. The median number of posts per active DGPRÄC member was 65 posts (IQR 15–160), corresponding to a median of 1.8 posts per month (IQR 0.6–4.0) since the first post. The median duration of Instagram use was around 3 years. Only a small minority had 2 Instagram accounts (10.6% of active members) or more than 2 accounts (4.1% of active members).

DGPRÄC members followed other Instagram accounts 2.6 times less often than they were followed themselves, with 653 median followers compared with 252 accounts followed. Around half of the accounts were used exclusively for work-related content (52.6%) and half both for work-related and private content (47.4%, Table 2). Professional posts mainly depicted the results of aesthetic procedures, primarily the results of breast augmentations and minimally invasive treatments such as Botox and hyaluronic acid injections. The results of nose surgery or liposuction for lipedema were less frequently posted. Professional posts occasionally showed pictures of the practice team members. Private posts mostly depicted hobbies, family, food, or landscapes.

**Table 2.** Descriptive Statistics of Instagram Activity (N = 407)

Variable	
No. total posts, median (IQR)	65 (15–160)
No. posts per month, median (IQR)	1.8 (0.6–4.0)
Time since first post, in mo, median (IQR)	37 (23–53)
No. accounts, n (%)	
1	347 (85.3)
2	43 (10.6)
>2	17 (4.1)
No. followers, median (IQR)	653 (308–1462)
No. accounts following, median (IQR)	252 (74–527)
Type of posts, n (%)	
Only work-related	214 (52.6)
Work-related and private	193 (47.4)

%, proportion of subjects.

**Fig. 2.**
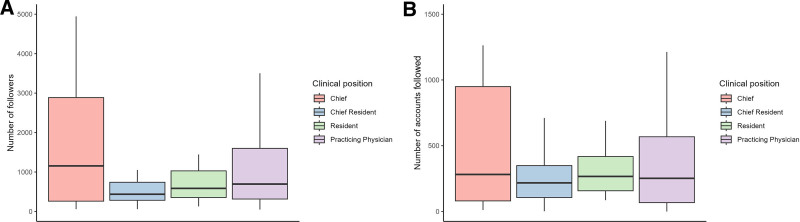
Instagram activity of DGRPÄC members categorized by clinical position. A, Box plots illustrating the distribution of the number of followers across different clinical positions. B, Box plots illustrating the distribution of the number of accounts followed across different clinical positions.

When comparing the number of followers to the number of accounts followed by clinical position, the ratio between these 2 numbers differed for the position of chiefs compared with the other clinical positions. The ratio of median followers versus median accounts followed ranged from 2 to 2.7 for attendings (437 versus 217), residents (585 versus 266), and practicing physicians (695 versus 252), whereas chiefs’ accounts were followed more than 4 times as often as they followed other accounts (1155 versus 281). For reasons of readability, outsiders were not shown in the box plots, as their inclusion would have excessively expanded the *y* axis range (Fig. 2). The maximum number of followers was 15,000, the maximum number of accounts followed was 7407; both values belonged to Instagram accounts of practicing physicians.

### Differences in Instagram Use With Respect to Clinical Position, Working Place, Sex, and Type of Federal State

**Fig. 3.**
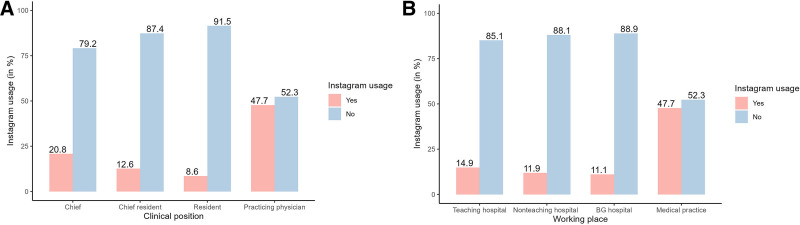
Percentage of Instagram use of DGPRÄC members categorized by clinical position and working place. A, Instagram use across different clinical positions. B, Instagram use across different working settings.

Almost 50% of practicing physicians (ie, 323–677) used Instagram to post work-related content. In contrast, plastic surgeons working in hospitals were significantly less likely to use Instagram for professional purposes. Among them, chiefs used Instagram most frequently (20.8%), followed by chief residents (12.6%) and residents (8.6%). The Pearson χ^2^ test of independence showed that there was a significant association between clinical position and Instagram use (χ^2^[3, N = 1281] = 173.4, *P* < 0.001) (Table 3, Fig. 3).

**Table 3.** Clinical Position Versus Instagram Use (N = 1281)

Clinical Position	Instagram Use, n (%)	No Instagram Use, n (%)
Chief	32 (20.8)	122 (79.2)
Attending	42 (12.6)	291 (87.4)
Resident	10 (8.6)	107 (91.5)
Practicing physician	323 (47.7)	354 (52.3)
Total	407 (31.8)	874 (68.2)

Pearson χ^2^ = 173.4; degrees of freedom = 3; *P* < 0.001.

A similar pattern emerged when examining Instagram activity with respect to workplace. Plastic surgeons working in private practice used Instagram at least 3 times more often (47.7%) compared with those in teaching hospitals (14.9%), nonteaching hospitals (11.9%), or BG hospitals (11.1%). The relationship between workplace and Instagram use was also significant (χ^2^[3, N = 1281] = 169.3, *P* < 0.001) (Table 4, Fig. 3).

**Table 4.** Working Place Versus Instagram Use

Working Place	Instagram Use, n (%)	No Instagram Use, n (%)
Teaching hospital	68 (14.5)	402 (85.5)
Nonteaching hospital	16 (11.9)	118 (88.1)
Medical practice	323 (47.7)	354 (52.3)
Total	407 (31.8)	874 (68.2)

Pearson χ^2^ = 168.6; degrees of freedom = 2; *P* < 0.001.

**Fig. 4.**
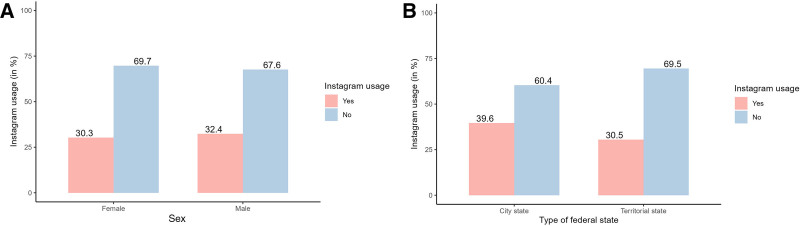
Percentage of Instagram use of DGPRÄC members categorized by sex and type of federal state. A, Instagram use across women and men. B, Instagram use across city states and territorial states.

Regarding sex, no significant difference in Instagram use between women and men was evident (χ^2^[1, N = 1281] = 0.6, *P* = 0.451). Although 115 of 380 women (30.3%) posted at least 1 work-related post on Instagram, 292 of 901 men (32.4%) used Instagram for professional purposes (Table 5, Fig. 4).

**Table 5.** Sex Versus Instagram Use (N = 1281)

Sex	Instagram Use, n (%)	No Instagram Use, n (%)
Female	115 (30.3)	265 (69.7)
Male	292 (32.4)	609 (67.6)
Total	407 (31.8)	874 (68.2)

Pearson χ^2^ = 0.5674; degrees of freedom = 1; *P* = 0.451.

Plastic surgeons working in 1 of the 3 German city-states used Instagram more frequently (39.6%) than those in the 13 German federal states (30.5%). Accordingly, the Pearson χ^2^ test indicated a significant association between the type of federal state and Instagram use (*χ*^*2*^ [1, N = 1281] = 5.9, *P* = 0.015) (Table 6, Fig. 4).

**Table 6.** Type of Federal State Versus Instagram Use (N = 1281)

Type of Federal State	Instagram Use, n (%)	No Instagram Use, n (%)
City state	72 (39.6)	110 (60.4)
Territorial state	335 (30.5)	764 (69.5)
Total	407 (31.8)	874 (68.2)

Pearson χ^2^ = 5.9362; degrees of freedom = 1; *P* = 0.015.

## DISCUSSION

We systematically examined the professional use of Instagram by plastic surgeons in Germany. Analyzing the data of 1281 members of the German specialist society DGPRÄC, we found that one-third of plastic surgeons posted professional content on Instagram. At the same time, about 44% of the members of the American Society of Plastic Surgeons had a professional Instagram account.^3^ Thus, German plastic surgeons might have been slightly less professionally engaged on Instagram compared with their American colleagues until 2022.

Almost half of the practicing physicians in our sample were professional Instagram users. In contrast, academic plastic surgeons were significantly less likely to use Instagram for professional purposes. These findings are consistent with previous research showing that plastic surgeons in private practice are more engaged on Instagram than academic plastic surgeons.^4,9^ In a competitive market, Instagram might be more suitable to increase their visibility, demonstrate their expertise in specific areas of plastic surgery, and attract prospective patients. Academic plastic surgeons tend to use other social media platforms such as LinkedIn or X (formerly Twitter).^4^ Although both academic and practicing surgeons use social media for education and networking, residents seem to place a stronger focus on self-promotion.^4^ In line with previous findings,^3^ a significantly higher proportion of professional Instagram users were located in metropolitan areas. Urbanized regions tend to have greater competition among medical professionals, including plastic surgeons. Thus, they might use social media to create a competitive advantage and market their services more effectively.

With less than 2 posts per month, the median activity was rather moderate. Furthermore, the median number of followers was merely 653. At the same time, American plastic surgeons presumably posted more frequently, and their accounts had more followers.^3^ On one hand, the German audience for plastic surgeons on Instagram might be smaller than the American audience. On the other hand, the number of followers depends on many factors, such as the type of posted content, marketing strategies, and the frequency of posting on Instagram. Thus, the comparably low number of followers in our sample might partly be explained by the relatively low frequency of posting new content.

Despite its advantages, it has been increasingly pointed out that the use of Instagram for medical purposes creates ethical and legal pitfalls. Although posts with patient-identifying content (eg, faces) are common on Instagram and can increase user engagement, publishing person-identifying features without specific permission is a violation of ethical conduct and can lead to high fines (eg, defined by the European General Data Protection Regulation). Therefore, written informed consent has to be obtained before publishing photographs or videos that could identify a specific person. Furthermore, taking photographs or videos must not distract the surgeon from performing a medical procedure, lengthen the procedure, or introduce unnecessary procedures for the purpose of creating social media content.^4,10^ To avoid distraction, taking photographs or recording videos should be performed by a third person. Further ethical issues include using filters or digital alterations, using unprofessional language, publishing inappropriate content for underage viewers, and posting sexualized content.^4,10^ Furthermore, the line between professional and personal content is often blurred, which may give patients the impression of a personal relationship. In our sample, almost half of the Instagram accounts depicted both work-related and private content. To keep their Instagram presence professional, surgeons have been advised to create separate professional and personal accounts and avoid interactions with patients on their personal accounts.^4^ Medical advice should not be given on social media, even when contacted on a professional account.^4^

When considering the legal background and ethical principles, plastic surgeons can make a meaningful contribution by sharing professional content on Instagram. On a largely unregulated platform, professional content improves the quality of medical information and contributes to reducing misinformation about surgical procedures.

Finally, we point out the limitations of our study. Because the data were collected in 2022, the results might have changed in a still growing and partly unpredictable world of social media. Referring to the latest publications, we assume that the number of plastic surgeons who professionally use Instagram has still been growing in recent years. Other results, such as the majority of Instagram users being practicing physicians or the focus on metropolitan areas, have probably changed less. Second, we did not systematically analyze the content that was published on the accounts. Reviewing the content, we found that the majority of professional posts depicted the results of aesthetic procedures, mainly breast augmentations. This finding was in line with previously published data from the United States indicating that nearly all Instagram posts from plastic surgeons depicted aesthetic procedures and female patients.^11^ Furthermore, we were not able to collect public engagement metrics (ie, number of likes, comments, saved posts, viewed reels, etc.). We are planning to collect more post-specific data in an updated version of this study.

## CONCLUSIONS

Plastic surgeons increasingly use Instagram to advertise their practices and attract prospective patients. About a third of German plastic surgeons used Instagram professionally in 2022, particularly in metropolitan areas. Practicing physicians were represented predominantly, indicating the perceived importance of the platform for visibility and patient acquisition in private practice. The relatively low frequency of posts and moderate number of followers indicate that they were not as active on Instagram and had a smaller reach compared with their American colleagues.

## DISCLOSURE

The authors have no financial interest to declare in relation to the content of this article.

## ACKNOWLEDGMENT

The authors acknowledge support from the Open Access Publishing Fund of the University of Tübingen.
